# Impact of a Modified Ultrasound-Guided Compression Protocol on Glue Migration in Endovenous Ablation Therapy: A Single-Centre, Prospective, Exploratory Pilot Comparative Cohort Study

**DOI:** 10.3390/jcm15166141

**Published:** 2026-08-07

**Authors:** Mohammed J. Alsaadi, Badr Aljabri, Abdulmajeed Altoijry, Abdulrahman M. Alfuraih

**Affiliations:** 1 Department of Radiology and Medical Imaging, College of Applied Medical Sciences in Al-Kharj, Prince Sattam Bin Abdulaziz University, Al-Kharj 11942, Saudi Arabia; m.alsaadi@psau.edu.sa; 2 Division of Vascular Surgery, Department of Surgery, College of Medicine, King Saud University, Riyadh 1322, Saudi Arabia; baljabri@ksu.edu.sa (B.A.); aaltoijry@kksu.edu.sa (A.A.)

**Keywords:** cyanoacrylate ablation, endovenous ablation, glue migration, saphenofemoral junction, ultrasound-guided compression, endovenous glue-induced thrombosis, great saphenous vein, varicose veins

## Abstract

**Background/Objectives:** Proximal glue migration toward the saphenofemoral junction (SFJ) and endovenous glue-induced thrombosis are recognised concerns during cyanoacrylate ablation. This exploratory pilot study compared standard and modified ultrasound-guided compression protocols. **Methods**: Thirty patients with incompetent great saphenous veins (GSVs) were allocated non-randomly, in two sequential cohorts, to a standard (*n* = 15) or modified (*n* = 15) protocol. The modified protocol added a “release-and-check” duplex assessment after the initial 3 min compression, a 1 min recompression, and an extra minute for veins > 5.5 mm. The primary outcome was glue migration distance from the compression level, measured immediately postoperatively. Clinical and thromboembolic outcomes were not systematically ascertained; follow-up was one week. **Results**: Baseline variables were comparable (standardised differences ≤ 0.31). Mean migration distance was lower with the modified protocol (0.80 ± 0.34 vs. 1.97 ± 0.51 cm; mean difference −1.17 cm, 95% CI −1.50 to −0.85; *p* < 0.0001), persisting after covariate adjustment. Residual stump length was greater (4.41 ± 0.34 vs. 2.49 ± 1.27 cm; *p* < 0.001), although this SFJ-referenced measure is confounded by the unrecorded compression distance. Deep venous extension occurred in 3/15 standard and 0/15 modified limbs (*p* = 0.22); all three were asymptomatic Cho grade III events, resolved at one week. Immediate migration was recorded in 7/15 versus 0/15 limbs (*p* = 0.006). **Conclusions**: In this small, non-randomised exploratory cohort, a “release-and-check” modification was associated with shorter immediate glue migration. These hypothesis-generating findings rest on an unblinded surrogate endpoint with one-week follow-up and do not establish clinical safety, effectiveness or durability; adequately powered randomised trials with blinded assessment and adjudicated endpoints are required.

## 1. Introduction

Chronic venous insufficiency with symptomatic varicose veins is common, impairs quality of life, and imposes a substantial healthcare burden [1,2]. Endovenous ablation techniques have largely replaced surgical stripping because they offer high occlusion rates, quicker recovery, and improve patient-reported outcomes [2,3,4,5]. Among non-thermal, non-tumescent options, cyanoacrylate endovenous ablation has gained widespread use because it avoids tumescent anaesthesia, shortens procedure time, and achieves occlusion rates comparable to those of thermal ablation [1,3,4,5,6].

Despite these advantages, cyanoacrylate ablation carries specific risks related to intraluminal glue behaviour, particularly proximal migration toward the saphenofemoral junction (SFJ) and extension into the deep venous system [7,8,9,10,11]. Proximal glue extension characterises endovenous glue-induced thrombosis (EGIT), which has been reported in 3–6% of treated limbs and, in rare cases, may progress to deep vein thrombosis or pulmonary embolism [7,12,13]. These findings emphasise the need for careful glue deployment and immediate post-delivery management of the proximal vein segment [13,14,15]. Ultrasound-guided external compression during and immediately after glue injection is commonly employed to enhance vein wall coaptation and minimise the risk of proximal glue spread [4,6,9,16]. However, suggested compression techniques, such as applying a fixed 3 min compression 5 cm below the SFJ, are mainly based on device instructions and expert opinion rather than direct comparative evidence [9,10,11,16]. Observational studies indicate that stump length and vein diameter are key factors influencing proximal extension, but there is no consensus on how to adjust compression duration, timing, or frequency based on these factors [7,11,13,16]. Current practice primarily employs an empirical “one-size-fits-all” compression method, which might not be ideal for high-risk veins. Variations in compression pressure, timing, or duration may lead to unintended proximal spread or incomplete vein occlusion, raising the risk of EGIT [7,10,11,12,13,17,18].

The goal of this study was to evaluate a proposed modified ultrasound-guided compression protocol in cyanoacrylate ablation of saphenous veins. This protocol contains a quick “release–check–recompress” cycle at the first treatment site and applies additional compression to larger veins. The working hypothesis was that this strategy would reduce proximal glue migration relative to the standard approach by improving immediate stump positioning and tailoring compression to vein size. This study also examined deep vein extension and early migration events in a prospective cohort of patients undergoing cyanoacrylate endovenous ablation.

## 2. Materials and Methods

### 2.1. Study Design

**Figure 1:**
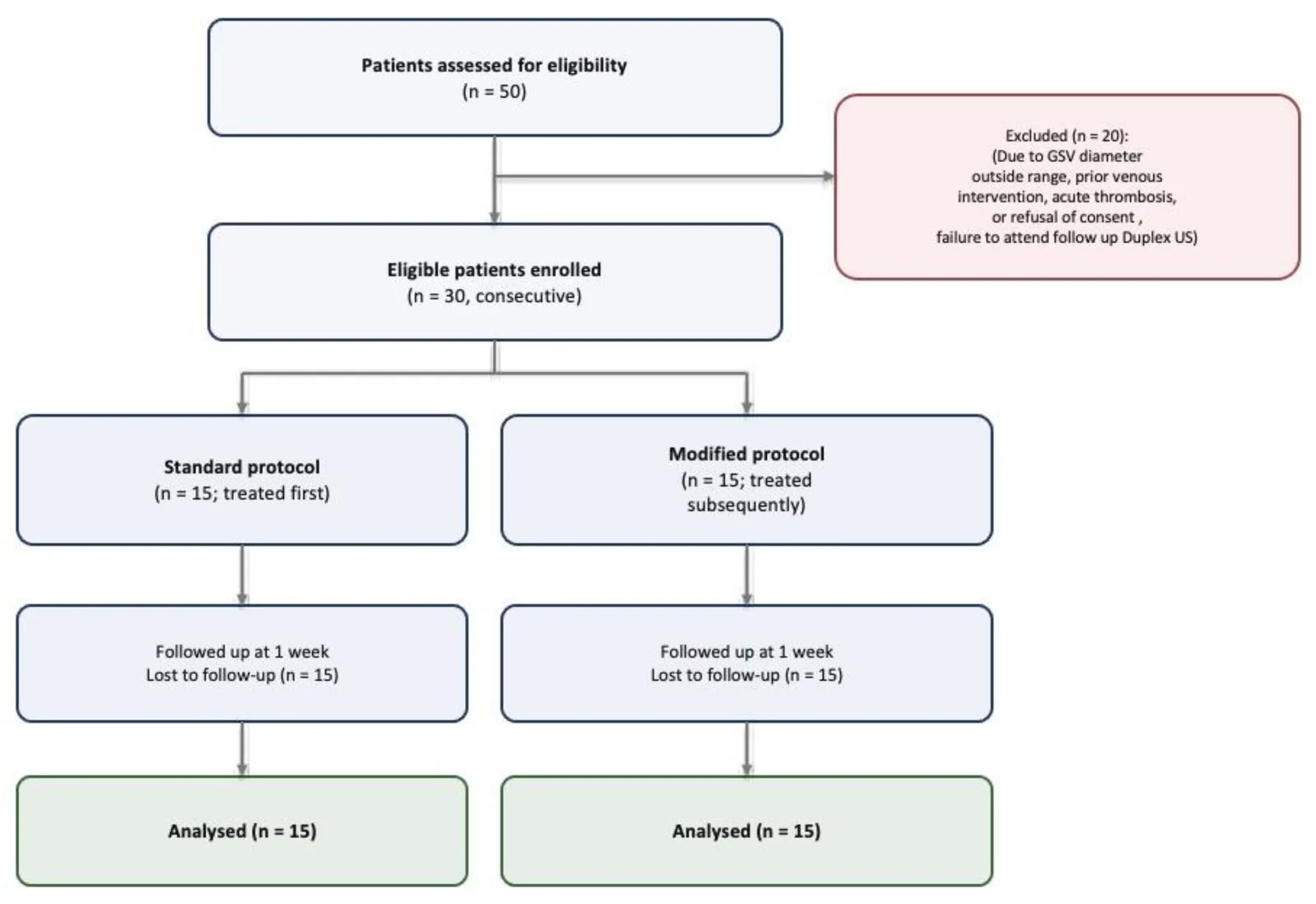
Study flow diagram. Of 50 patients assessed for eligibility, 20 were excluded, and 30 were enrolled and allocated to the standard (*n* = 15) or modified (*n* = 15) protocol. All 30 completed a one-week follow-up and were analysed.

In accordance with the STROBE statement for observational studies, we conducted a single-centre, prospective, exploratory pilot comparative cohort study of a modified ultrasound-guided compression protocol during cyanoacrylate endovenous ablation of the great saphenous vein (GSV) (Supplementary Material S1). No formal a priori sample size calculation was performed: the sample of 15 limbs per group was determined by feasibility, and the study was designed and is reported as a hypothesis-generating pilot restricted to immediate technical and anatomical ultrasound observations. It was not designed or powered to evaluate clinical effectiveness, durability of occlusion, or clinical safety. The protocol was ratified by the Institutional Review Board (IRB approval RHPT/026/016; see the Institutional Review Board Statement). All procedures were conducted in accordance with the Declaration of Helsinki, and written informed consent was obtained from each participant. Thirty consecutive eligible patients were enrolled from 50 patients assessed for eligibility; 20 were excluded because of a GSV diameter outside the specified range, prior venous intervention, acute thrombosis, failure to attend the follow-up duplex ultrasound, or refusal of consent (Figure 1). The 30 eligible patients were allocated, in two sequential (non-randomised) cohorts, to the standard protocol (*n* = 15, treated first; enrolled between 29 April 2026 and 15 May 2026) or the modified protocol (*n* = 15, treated subsequently; enrolled between 15 May 2026 and 2 June 2026). Both cohorts were treated by the same two vascular surgeons, each with more than 15 years of experience in endovenous ablation; the distribution of cases between operators was *n* = 8 and *n* = 7 in the standard cohort and *n* = 7 and *n* = 8 in the modified cohort. Duplex assessments were performed by two sonographers who likewise worked across both arms. Because the operators were experienced and their learning curve had plateaued before the study began, an intra-study learning effect is an unlikely explanation for the observed differences; however, sequential rather than randomised allocation means that temporal confounding, selection bias, procedural drift and unmeasured confounding cannot be excluded, and the two cohorts were treated in different calendar periods. Clinical and ultrasound examinations were performed immediately after the procedure and at one-week follow-up. For planning a confirmatory trial, detecting the observed reduction in immediate post-procedural migration with 80% power at a two-sided α of 0.05 would require approximately 14 limbs per group, whereas detecting a clinically meaningful reduction in a rare thrombotic event such as deep-vein extension (e.g., from 6% to 1%) would require on the order of 180 limbs per group; an adequately powered trial should therefore be sized primarily for clinically adjudicated safety endpoints.

Eligibility criteria were (a) symptomatic superficial venous disease with duplex-confirmed axial GSV reflux (reflux duration > 0.5 s); (b) clinical CEAP class C1 or C2; (c) GSV diameter of 3.8 to 6.5 mm; and (d) age > 18 years. Exclusion criteria were prior surgery on the vein in the target limb, active deep vein thrombosis, prior pulmonary embolism, known contraindication to cyanoacrylate, or glue allergy. All included patients had duplex-confirmed axial GSV reflux and were offered truncal ablation on the basis of symptomatic axial reflux rather than the visible surface class. Patients classified as CEAP C1 had only reticular veins or telangiectasias on surface examination; the symptoms attributed to axial reflux in these patients were leg pain on standing, heaviness, throbbing, night cramps and leg oedema, elicited by a structured history taken from each participant. Alternative causes were excluded clinically by musculoskeletal, arterial, neurogenic and dermatological assessment. No trial of conservative management with graduated compression was undertaken before ablation; this departure from the usual stepped-care pathway is acknowledged in the Limitations section. The CEAP classification of every patient was re-verified against the source clinical records during this revision. We acknowledge that truncal ablation in C1 limbs is not uniformly endorsed by current guidelines, which reserve their strongest recommendations for C2 disease and above, and that the high proportion of C1 patients (70%) in this cohort materially limits the generalisability of the findings; this is stated in the Limitations section.

### 2.2. Treatment Protocol

**Figure 2:**
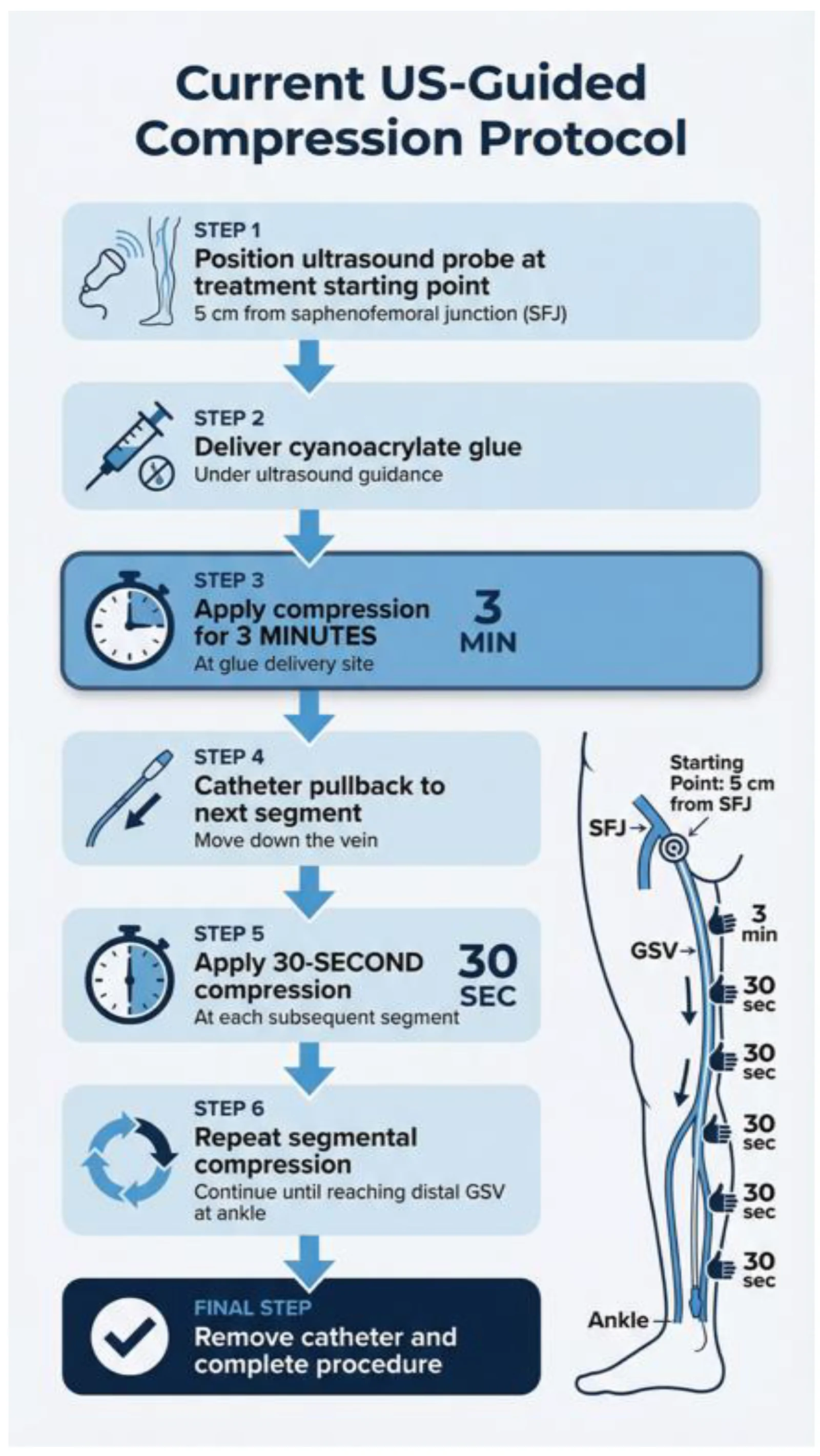
Schematic of the standard cyanoacrylate endovenous ablation protocol, showing probe placement 5 cm distal to the saphenofemoral junction (SFJ) and the standard 3 min initial compression followed by sequential 30 s segmental compressions during catheter pullback.

All procedures were performed using the VenaSeal system (Medtronic, Dublin, Ireland) under continuous ultrasound guidance, and the two groups differed only in the compression strategy applied at the first treatment site. To avoid ambiguity, four distinct positions are defined here. (i) The catheter-tip position for the first injection was 5 cm distal to the SFJ, confirmed on longitudinal duplex imaging. (ii) The glue-delivery point was the same level, since cyanoacrylate was delivered from the catheter tip. (iii) The ultrasound-transducer position was transverse, directly over that level. (iv) The compression level was the level at which external manual compression was transmitted through the transducer, i.e., nominally the same 5 cm point. In the standard technique, cyanoacrylate was delivered under continuous ultrasound visualisation, and compression was then maintained continuously over the glue-delivery point for 3 min. The catheter was withdrawn to the next venous segment, where compression was applied for 30 s; segmental compressions were repeated until the distal GSV at the level of the ankle was treated, after which the catheter was removed (Figure 2). The distance from the SFJ at which compression was applied and verified sonographically at the time of the procedure was not recorded as a numerical variable in the case-report form.

**Figure 3:**
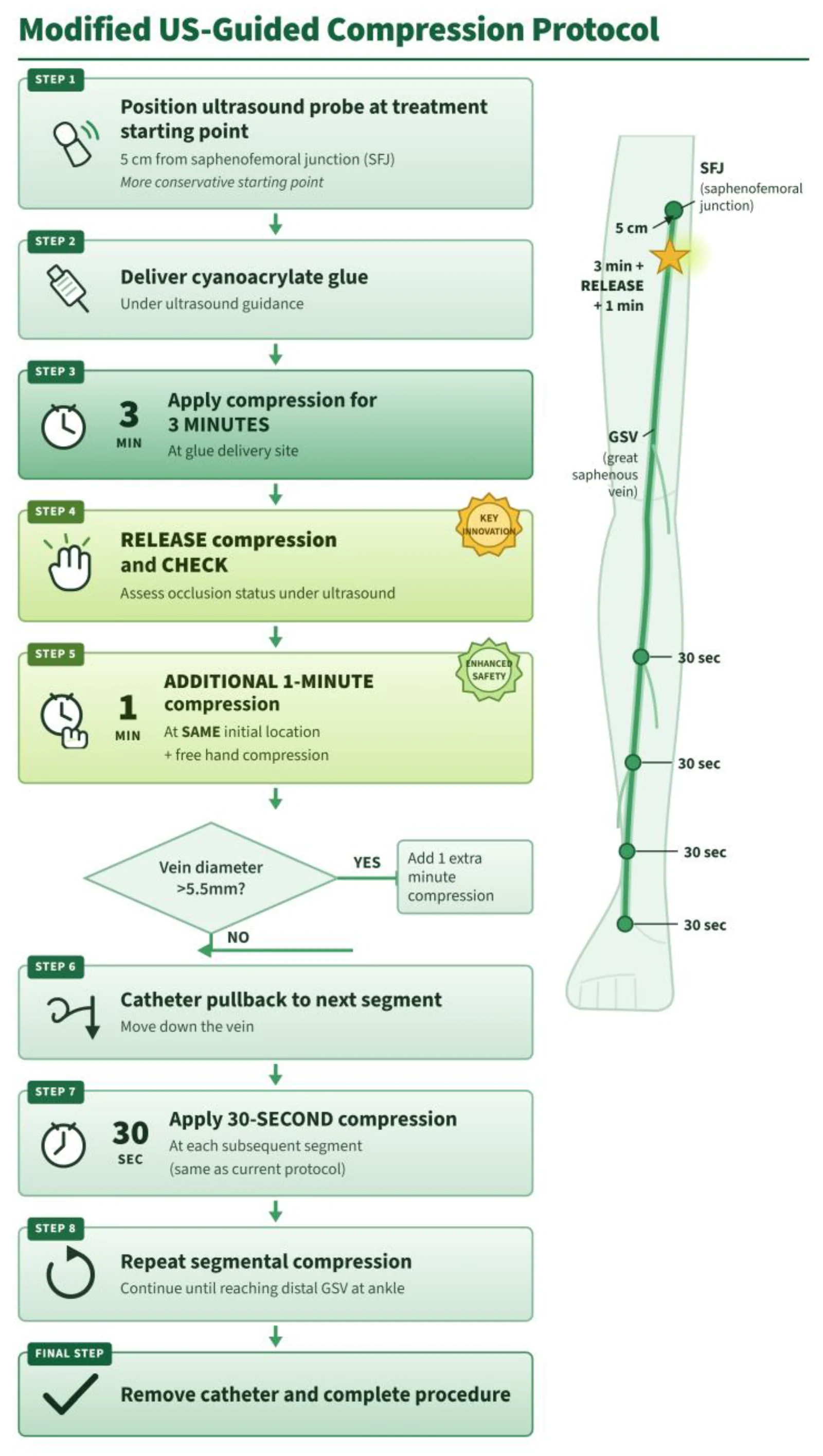
Schematic of the modified “release-and-check” compression protocol, illustrating the three additional manoeuvres applied at the initial treatment site: release and sonographic assessment after the initial 3 min compression; 1 min recompression at the same site; and an additional 1 min compression for veins > 5.5 mm.

The modified protocol comprised the same basic steps plus three additional manoeuvres performed at the first treatment site (Figure 3). After the initial 3 min compression at the starting point (5 cm from the SFJ), compression was released, and the occlusion status was assessed sonographically (release-and-check). Free-hand compression was then reapplied at the same site for 1 min. A diameter-based criterion was also applied: when the GSV diameter at the treatment site exceeded 5.5 mm, one extra minute of compression was delivered at the initial point. Subsequent catheter withdrawal used 30 s segmental compression, identical to the standard protocol. The relationship to the manufacturer’s Instructions for Use (IFU) was as follows: the VenaSeal IFU specifies delivery of two 0.1 mL aliquots 1 cm apart beginning 5 cm distal to the SFJ, with compression applied over the treated segment for 3 min at the first site and 30 s at subsequent sites during sheath and catheter withdrawal. The release-and-check assessment, the 1 min recompression and the diameter-based additional minute are all applied after the IFU-specified 3 min compression has been completed at the first site; they therefore add to, and do not replace, shorten or contradict, the manufacturer-recommended technique. The complete modification, including the diameter-based criterion (a diameter-based additional minute), was specified in the study protocol before the first patient in the modified cohort was treated and was included in the protocol approved by the Institutional Review Board. It should be emphasised that the modification is a bundle of three co-administered components; the present design cannot determine the contribution of any individual component.

### 2.3. Outcome Measures

The primary outcome was the glue migration distance, defined as the displacement of the proximal edge of the echogenic glue cast from the level at which compression had been applied, measured in centimetres as a continuous variable on duplex ultrasound immediately after the procedure (within 5 min of catheter removal). Measurements were made in the longitudinal plane with electronic callipers, from the proximal margin of the glue cast to the skin marking placed over the compression level at the start of the procedure; a single measurement per limb was recorded. Because this outcome is referenced to the compression level rather than to the SFJ, it is arithmetically independent of the distance between the compression level and the SFJ. The principal anatomical safety measure was residual stump length, defined as the distance from the proximal glue edge to the SFJ at the final measurement; unlike the primary outcome, this measure is referenced to the SFJ and is therefore influenced by the level at which compression was applied. Secondary outcomes were (a) extension into the deep venous system, defined as any glue or thrombus extending beyond the SFJ into the common femoral vein (CFV) on duplex imaging (Yes/No); (b) immediate post-procedural migration (Yes/No); and (c) migration at the one-week follow-up visit (Yes/No). Every event of deep venous extension was graded according to the classification of Cho et al. [7], which grades EGIT by the proportion of the deep venous luminal area occupied by glue or thrombus: grade I < 25%, grade II 25–49%, grade III 50–74% and grade IV 75–100%. Because this classification is based on cross-sectional luminal area rather than linear extent, the length of protrusion into the CFV was not recorded; the proportion of the CFV luminal area involved is reported for every event in the results section. The two binary migration outcomes were recorded prospectively as qualitative duplex judgements: the presence or absence of visible proximal displacement of the glue cast relative to the intended compression level at the immediate post-procedural scan, and any further proximal displacement between that scan and the one-week scan, and were not derived by dichotomising the continuous migration measurement. The continuous and binary endpoints are therefore related but not algebraically dependent: a group mean migration distance greater than zero is compatible with an event count of zero, and the two classifications do not coincide in every individual limb. To make this relationship explicit rather than leave it implicit, a post hoc sensitivity analysis in which immediate migration is instead defined as a continuous displacement of ≥ 2.0 cm is reported in Section 3.3. All measurements were performed live, during or immediately after the procedure, by two experienced sonographers using a standardised duplex protocol. Because measurements were obtained during the procedure by the treating team, the assessors were aware of the treatment allocation and were not blinded, and neither interobserver nor intraobserver reliability was quantified; intraclass correlation coefficients could not be calculated because ultrasound clips and still images were not retained for offline re-measurement. No claim is therefore made regarding the precision, reproducibility or internal consistency of these centimetre-scale measurements, and measurement bias in favour of the modified protocol cannot be excluded.

### 2.4. Statistical Analysis

Data were analysed using SPSS version 29 (IBM Corp., Armonk, NY, USA), with confirmatory computation in Python 3.12 (SciPy 1.14, statsmodels 0.14). Normality was assessed with the Shapiro–Wilk test. It was satisfied for the primary outcome in both groups (*p* = 0.53 and *p* = 0.94) but not for residual stump length in the standard group (*p* = 0.005), which was bimodal because of the three limbs with deep venous extension; for that outcome, the Mann–Whitney U test is reported alongside the *t*-test. Continuous variables are presented as mean ± standard deviation and were compared using independent-samples *t*-tests. The primary outcome was additionally examined by analysis of covariance (ANCOVA), first adjusting for GSV diameter alone and then adjusting for GSV diameter, age, body mass index and smoking status, with adjusted mean differences and 95% confidence intervals (CIs) reported, and by a non-parametric Mann–Whitney U test as a sensitivity analysis. Categorical variables are presented as counts (percentages) and were compared using Fisher’s exact test; risk differences with 95% CIs are reported for all binary outcomes, and Wilson score 95% CIs are reported for every individual proportion. Given the small sample, baseline balance was assessed using standardised differences in addition to *p* values. A post hoc sensitivity analysis re-derived the immediate migration endpoint from the continuous measurement using a ≥2.0 cm threshold. The inferred compression level reported in Section 3.1 is derived arithmetically as the sum of two post-procedural outcome measurements (residual stump length plus migration distance); it is therefore not an independently measured baseline covariate, and adjusting the migration or stump outcomes for a quantity computed from those same outcomes would be circular. It was consequently not used as a covariate in any adjusted model, and this is treated as an unresolved limitation rather than as something the analysis can correct. No formal treatment-by-diameter interaction test was performed, and no diameter-stratified subgroup analysis is presented, because the stratum of veins > 5.5 mm contained only eight limbs in total. Statistical significance was defined as *p* < 0.05; given the exploratory design and multiple endpoints, *p* values are interpreted descriptively and no multiplicity adjustment was applied.

## 3. Results

### 3.1. Baseline Characteristics

Thirty patients were enrolled and equally allocated to the standard protocol (*n* = 15) or the modified protocol (*n* = 15). Baseline characteristics are summarised in Table 1. The cohorts were comparable for every variable recorded: age (46.0 ± 7.9 vs. 47.6 ± 7.7 years; standardised difference 0.21), sex (5 vs. 4 men; 0.15), body mass index (25.5 ± 3.0 vs. 26.3 ± 2.0 kg/m^2^; 0.31), GSV diameter (5.10 ± 0.80 vs. 5.17 ± 0.82 mm; 0.09), the proportion of veins > 5.5 mm (5 vs. 3 limbs; 0.30), vein depth (2.19 ± 0.30 vs. 2.19 ± 0.33 cm; 0.02), CEAP class (5 vs. 4 limbs C2; 0.15), treated limb (0.13), smoking (5 vs. 4 patients; 0.15) and diabetes (3 vs. 4 patients; 0.16). No patient in either cohort had thrombophilia or a history of superficial thrombophlebitis, and no patient in either cohort was taking antiplatelet or anticoagulant therapy at baseline. All standardised differences were therefore ≤0.31, the largest being for body mass index and for the proportion of large veins, and no variable differed significantly. This apparent similarity should not be over-interpreted: with 15 limbs per group, this study has very little power to detect baseline imbalance, and sequential non-randomised allocation leaves unmeasured factors free to differ between cohorts. Variables that were not recorded, and therefore could not be compared, are treated vein length, vein tortuosity, reflux time and peak reflux velocity, adjunctive procedures and post-procedure mobilisation. In both cohorts, the initial compression was nominally delivered 5 cm distal to the SFJ per protocol, but the actual distance was not documented numerically in the case-report form. A post hoc inferred compression level, calculated as the sum of residual stump length and migration distance, was more distal in the modified cohort (5.21 ± 0.33 vs. 4.47 ± 0.88 cm; *p* = 0.005). The distribution of this derived quantity is informative: in the modified cohort it ranged from 4.9 to 6.0 cm, consistent with the nominal 5 cm protocol, whereas in the standard cohort it ranged from 3.1 to 5.7 cm, with four limbs yielding values of 3.1–3.4 cm that are implausibly proximal for a protocol-adherent starting point. Those four limbs include all three with deep venous extension. This pattern is compatible with two explanations that the present data cannot distinguish: genuine variation in where compression was applied, or measurement error in one or both component measurements in limbs where the glue cast lay close to the SFJ. Either way, the derived quantity is not an independent baseline covariate; it is reported as a descriptive post hoc observation only, and it has been excluded from Table 1. It follows that the between-group differences in residual stump length and in proximity to the SFJ cannot be attributed to the intervention alone.

### 3.2. Primary Outcome: Glue Migration Distance

**Figure 4:**
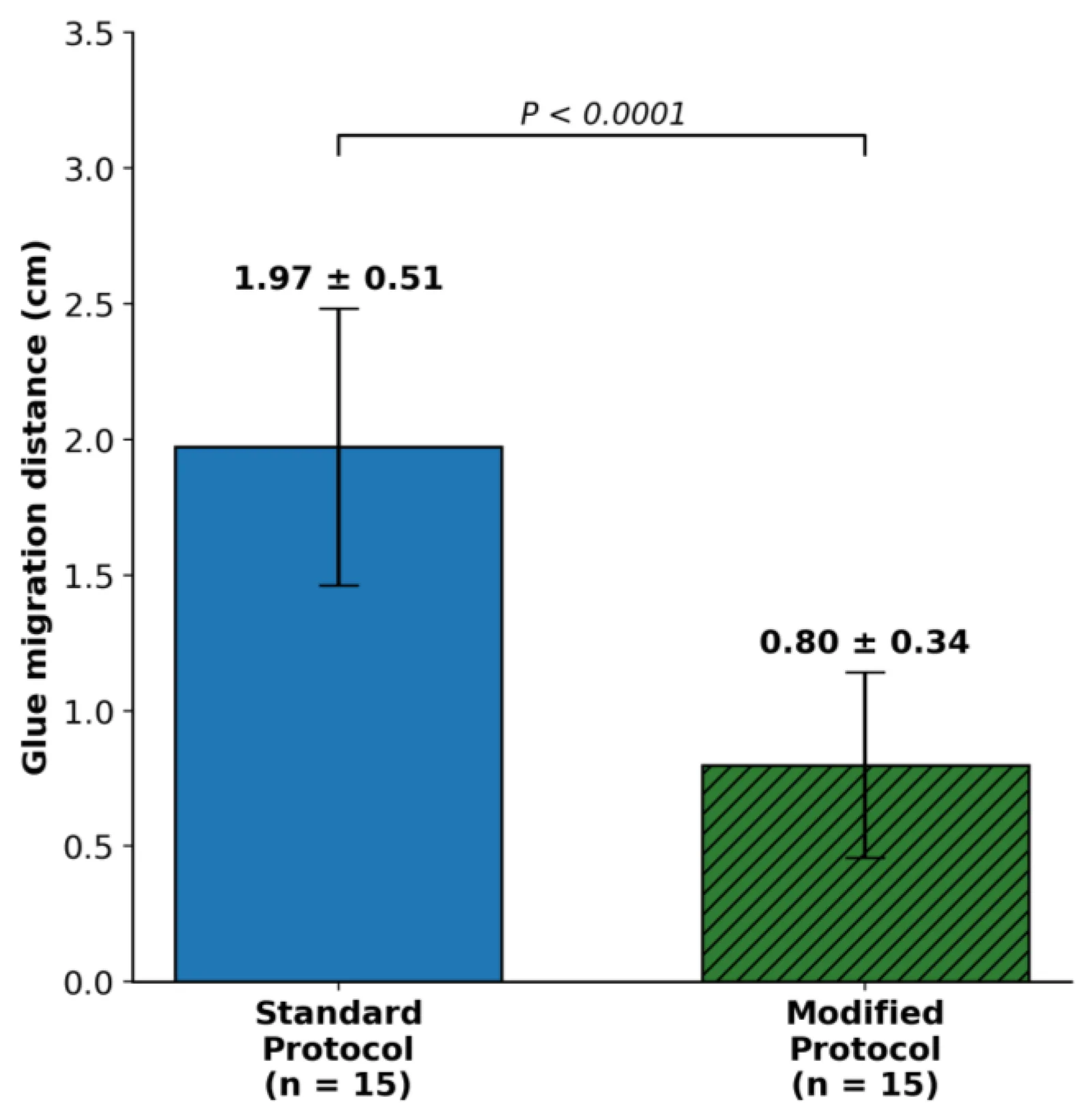
Bar graph (mean ± standard deviation) comparing glue migration distance, measured from the compression level, between the standard and modified protocols (1.97 ± 0.51 cm vs. 0.80 ± 0.34 cm; *p* < 0.0001).

The mean glue migration distance, measured from the compression level, was lower with the modified protocol (Figure 4). The standard protocol produced a mean migration distance of 1.97 ± 0.51 cm (95% CI, 1.69 to 2.26; range 1.20 to 2.90) compared with 0.80 ± 0.34 cm (95% CI, 0.61 to 0.99; range 0.20 to 1.40) in the modified protocol—a mean difference of −1.17 cm (95% CI −1.50 to −0.85; *p* < 0.0001; Cohen’s d = 2.70; Mann–Whitney U *p* < 0.001). Because migration was referenced to the compression level rather than to the SFJ, this particular comparison is arithmetically independent of the level at which compression was applied, although it remains subject to the unblinded measurement and non-randomised allocation described above. The difference persisted after adjustment for GSV diameter (ANCOVA adjusted difference −1.20 cm; 95% CI, −1.47 to −0.92; *p* < 0.0001) and after further adjustment for age, body mass index and smoking status (adjusted difference −1.19 cm; 95% CI, −1.44 to −0.94; *p* < 0.0001). Residual stump length (the distance of the proximal glue edge from the SFJ) was greater in the modified group (4.41 ± 0.34 vs. 2.49 ± 1.27 cm; mean difference +1.92 cm, 95% CI +1.22 to +2.62; *p* < 0.001; Mann–Whitney U *p* < 0.001; adjusted for GSV diameter, +1.97 cm, 95% CI +1.39 to +2.55). As noted in Section 3.1, this SFJ-referenced measurement is influenced by the apparent between-cohort difference in the level at which compression was applied and cannot be attributed to the intervention alone. Diameter-stratified data are not presented, because the stratum of veins > 5.5 mm contained only five standard and three modified limbs; the absolute between-group differences in mean migration were similar in the two strata (−1.07 cm for veins ≤ 5.5 mm and −1.17 cm for veins > 5.5 mm), no formal treatment-by-diameter interaction was tested, and these data do not demonstrate effect modification by vein diameter.

### 3.3. Secondary Outcomes

**Figure 5:**
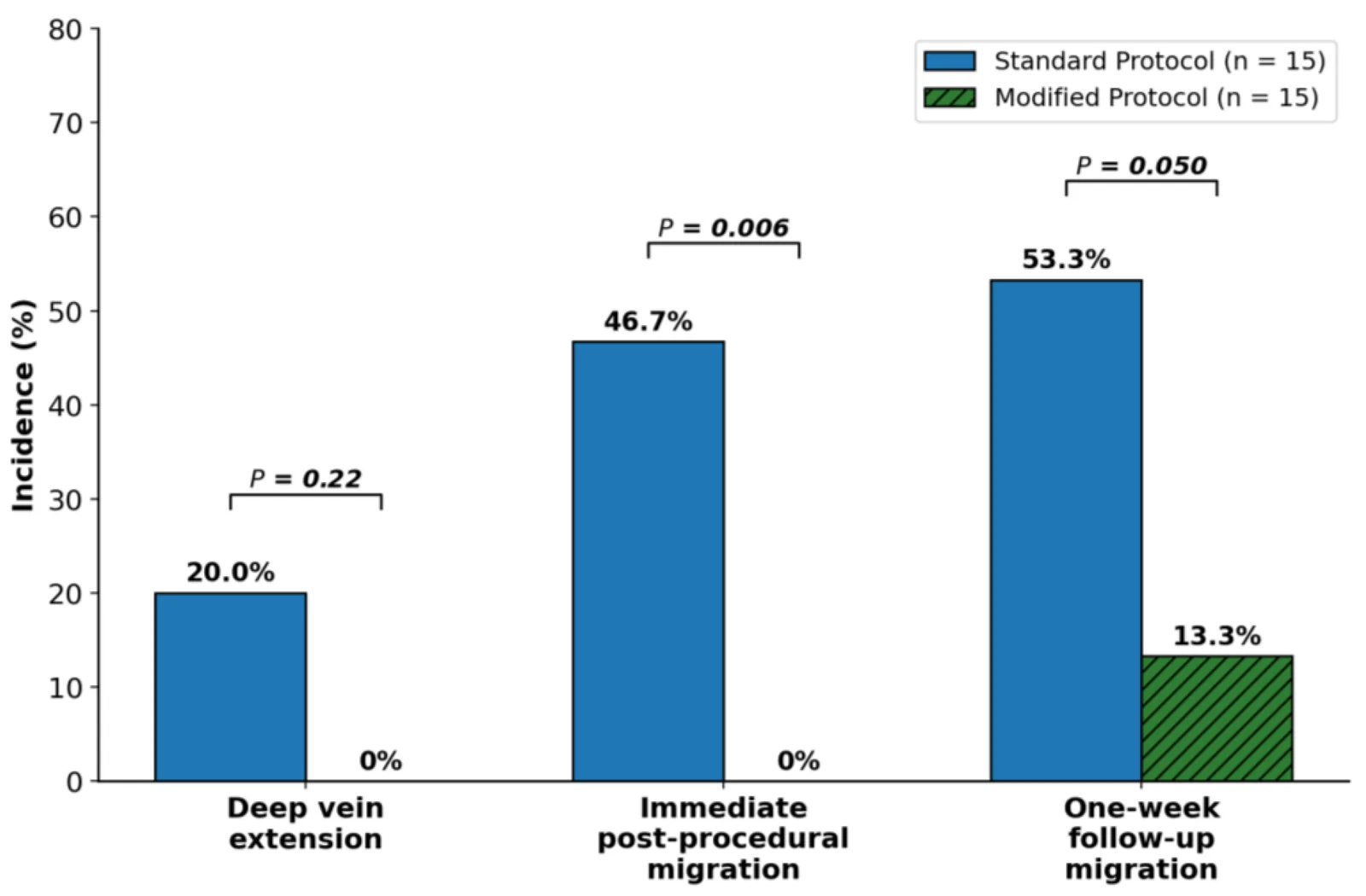
Bar graph of secondary outcomes (deep venous extension, immediate post-procedural migration, and one-week migration) in the standard and modified protocol groups.

Secondary outcomes are detailed in Table 2 and Figure 5. Extension into the deep venous system was observed in 3 of 15 limbs (20.0%; Wilson 95% CI 7.0 to 45.2) in the standard group and in none (0.0%; Wilson 95% CI 0.0 to 20.4) of the modified group (risk difference −20.0%, 95% CI −40.2 to +0.2; *p* = 0.22, Fisher’s exact test). Because the confidence interval for this risk difference includes zero, the comparison does not establish any difference in safety, and the absence of observed events in the modified group is an exploratory observation only. Individual details of the three affected limbs are given in Table 3. All three occurred in the largest veins treated in that cohort (GSV diameter 6.0, 6.2 and 6.3 mm), all were in CEAP C2 limbs, and all were asymptomatic, detected on protocol duplex rather than through clinical presentation. On retrospective adjudication, all three were Cho grade III (50–74% of the CFV luminal area), with 50%, 70% and 60% luminal involvement, respectively, and all three had resolved on duplex imaging at one week. The 20.0% incidence in the standard cohort is higher than the 3–6% generally reported for EGIT, and the grade distribution is more severe than in the series of Cho et al., in which no grade III or IV event occurred among 11 EGITs [7]. This should be interpreted in the light of the small denominator (the confidence interval extends from 7% to 45%), the high proportion of large veins in that cohort, the apparently more proximal compression level in those particular limbs (Section 3.1), and the fact that every duplex-detected extension beyond the SFJ was counted. Immediate post-procedural migration occurred in 7 of 15 limbs (46.7%; Wilson 95% CI 24.8 to 69.9) in the standard group compared with none (0.0%; Wilson 95% CI 0.0 to 20.4) in the modified group (risk difference −46.7%, 95% CI −71.9 to −21.4; *p* = 0.006). In the prespecified sensitivity analysis in which this endpoint was instead derived from the continuous measurement using a ≥2.0 cm threshold, the corresponding figures were 6 of 15 (40.0%; Wilson 95% CI 19.8 to 64.3) versus 0 of 15 (0.0%; Wilson 95% CI 0.0 to 20.4) (risk difference −40.0%, 95% CI −64.8 to −15.2; *p* = 0.017); the two definitions therefore agree in direction and magnitude but do not classify every individual limb identically, which is why both are reported. At one-week follow-up, migration was observed in 8 of 15 limbs (53.3%; Wilson 95% CI 30.1 to 75.2) in the standard group versus 2 of 15 (13.3%; Wilson 95% CI 3.7 to 37.9) in the modified group (risk difference −40.0%, 95% CI −70.6 to −9.4; *p* = 0.050); given the borderline *p* value and the small sample, this difference should not be overinterpreted, and the one-week assessment is a single descriptive time point rather than evidence of a durable effect. Immediate technical occlusion was complete in all 30 treated limbs (15/15 in each group). Symptomatic phlebitis, hypersensitivity or foreign-body reactions, deep vein thrombosis, pulmonary embolism, symptom improvement, the Venous Clinical Severity Score (VCSS) and quality-of-life measures were not prospectively or systematically ascertained, and no structured adverse-event surveillance was undertaken; the absence of recorded events therefore cannot be interpreted as an absence of events, and no statement about the overall safety profile of either protocol can be made from these data.

### 3.4. Descriptive Distance Categories

**Figure 6:**
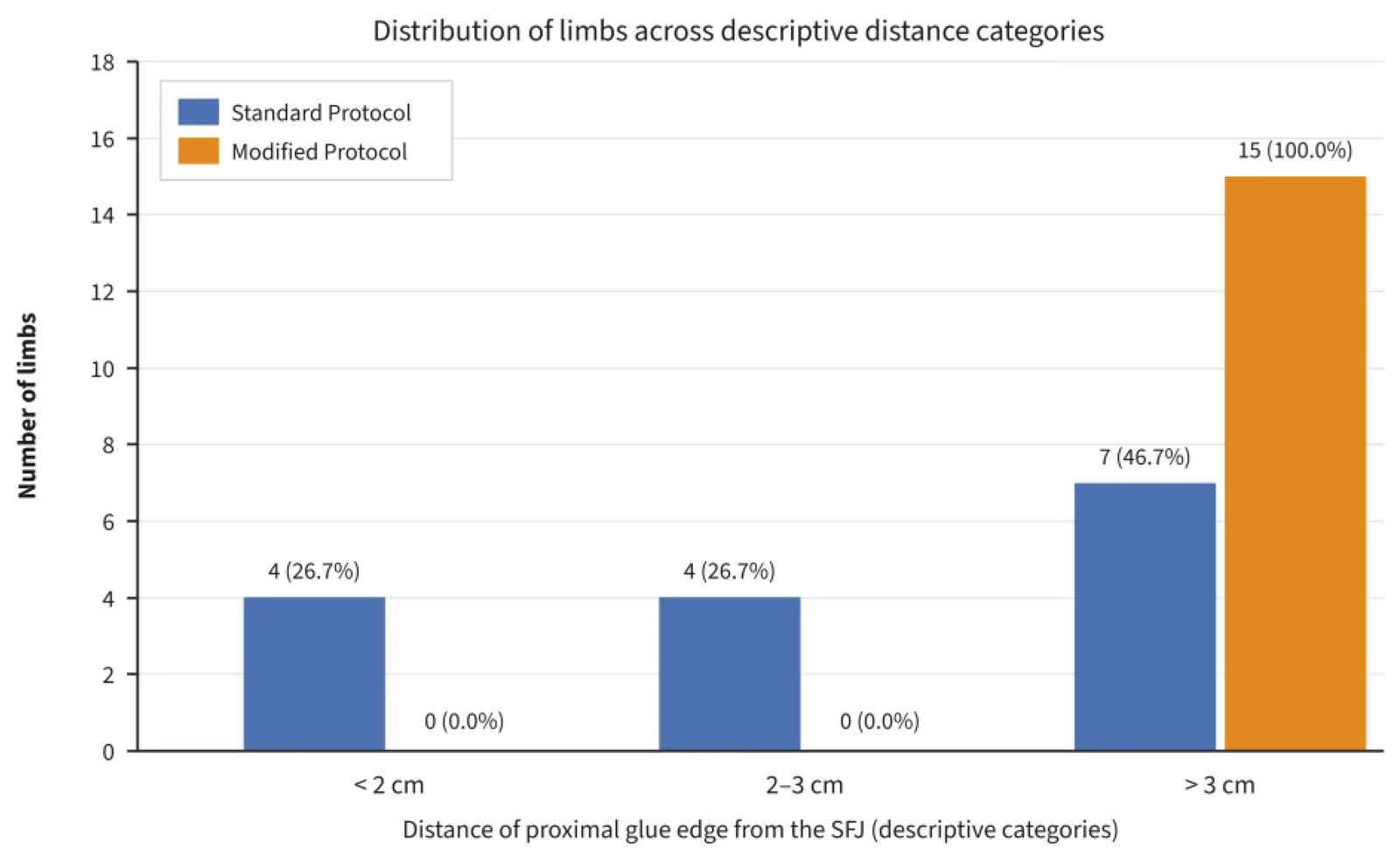
Distribution of limbs across descriptive distance categories (<2 cm, 2–3 cm, >3 cm from the SFJ) for the standard and modified protocols. The categories are descriptive only and do not represent validated clinical thresholds.

To describe how far the glue column lay from the SFJ at the final measurement, limbs were grouped into three neutral, purely descriptive distance categories: <2 cm, 2–3 cm, and >3 cm. These are arbitrary descriptive strata chosen for readability; they are not validated clinical thresholds, have not been shown to correspond to any level of thrombosis risk, and no risk label, safety label, or clinical interpretation is attached to them. The distribution is shown in Table 4 and Figure 6. Under the standard protocol, 4 of 15 limbs (26.7%) fell in the <2 cm category, 4 of 15 (26.7%) in the 2–3 cm category, and 7 of 15 (46.7%) in the >3 cm category; under the modified protocol, all 15 limbs (100%) fell into the >3 cm category. This distribution is a direct restatement of the residual stump length data and is therefore subject to the same caveat: it is influenced by the apparent between-cohort difference in the level at which compression was applied and does not by itself demonstrate any clinical protection.

## 4. Discussion

In this exploratory pilot cohort, adding a short release–check–recompress cycle at the first treatment site during cyanoacrylate endovenous ablation was associated with a shorter immediate glue migration distance (mean difference −1.17 cm; Cohen’s d = 2.70), and the difference persisted after adjustment for vein diameter, age, body mass index and smoking. Because migration was measured relative to the compression level, this comparison is arithmetically independent of the starting position; it is not, however, independent of the non-randomised, sequential allocation or of the unblinded, live measurement of a centimetre-scale ultrasound variable, both of which could bias the estimate. Baseline comparability on the variables we recorded is weak reassurance in a sample of this size and does not establish exchangeability of the cohorts. Residual stump length was greater in the modified group (4.41 vs. 2.49 cm), but this SFJ-referenced measurement is confounded by the apparent between-cohort difference in the level at which compression was applied and cannot be attributed to the intervention alone. Extension into the deep venous system was recorded in 3 of 15 standard limbs and in none of the modified limbs; with a risk-difference confidence interval that includes zero (*p* = 0.22), no difference in safety is established. All three events were asymptomatic, were graded Cho III, and had resolved at one week, so although they were radiologically substantial, they were clinically silent and self-limiting within the observation window. These findings are hypothesis-generating and should not be read as evidence that the modified protocol is safer.

The one-week assessment is reported descriptively. Migration was recorded in 8 of 15 standard limbs and in 2 of 15 modified limbs at that visit. A single follow-up scan at seven days cannot establish durability, stability or longer-lasting glue positioning, and no such claim is made; stump length after cyanoacrylate closure is known to change over subsequent months [13,19], and delayed thrombotic changes at the SFJ have been reported after cyanoacrylate and other non-thermal endovenous procedures [3,4,5,6,7,12,13,14,15]. Whether any early difference in glue position persists beyond one week is unknown from these data.

The relationship between vein diameter and glue behaviour remains unresolved in the literature and is not clarified by the present data. Larger GSV diameter has been associated with more advanced venous disease and with thrombotic complications after non-thermal, non-tumescent ablation in some series [11,12,13], whereas Cho et al. identified a smaller saphenous diameter (<5 mm) as a risk factor for endovenous glue-induced thrombosis, and Park and Kim reported that a larger GSV diameter was associated with shorter glue extension and a longer remnant stump [7,13,17]. Our three events occurred in the largest veins we treated (6.0–6.3 mm), which is the opposite of the association reported by Cho et al.; with three events, this observation carries no weight beyond noting the inconsistency in the literature. Our study included only eight limbs with a diameter above 5.5 mm, tested no-treatment-by-diameter interactions, and found absolute between-group differences in mean migration that were similar in the two diameter strata; accordingly, no diameter-stratified analysis is presented, and no diameter-based recommendation can be derived from this work. It should also be noted that the modified protocol is a bundle of three co-administered components—release-and-check imaging, routine recompression, and an additional minute for larger veins—so the contribution of any individual component, including the diameter-based criterion, cannot be determined from this design. The demographic profile of patients treated for varicose veins also varies by region, which may affect generalisability. In a Balkan cohort from Albania, varicose-vein patients were predominantly middle-aged (41–65 years), largely female, frequently overweight, and mostly CEAP C2 [20]; epidemiological data indicate a substantially lower prevalence of varicose veins and chronic venous insufficiency in East and South-East Asian populations than in Western or Mediterranean populations [21]. Our single-centre Middle Eastern cohort, in which 70% of treated limbs were CEAP C1 and in which no patient underwent a trial of conservative management before ablation, sits outside the population and the care pathway in which truncal ablation is most commonly performed, and this materially limits extrapolation. Finally, the local thrombotic complications of endovenous ablation span a recognised spectrum—including endothermal and endovenous glue-induced thrombosis, superficial thrombophlebitis and, rarely, deep vein thrombosis or pulmonary embolism—which we summarise in Table 5 to contextualise the deep venous extension events observed here.

The distribution of limbs across the descriptive distance categories mirrors the residual stump length data: under the standard protocol 26.7% of limbs ended with the proximal glue edge less than 2 cm from the SFJ, whereas under the modified protocol all limbs ended more than 3 cm from the SFJ. We emphasise that these categories are descriptive strata only. They were not prospectively validated, no threshold has been shown to correspond to a defined thrombosis risk, and the distribution is influenced by the apparent between-cohort difference in the level at which compression was applied. They are therefore presented without safety labels and should not be read as identifying a protective or clinically acceptable range.

Several mechanisms could plausibly account for the shorter measured migration distance, although none are tested by this study. The release-and-check step allows sonographic assessment of occlusion and glue position before the catheter is withdrawn, in principle permitting recognition of residual flow or early proximal displacement [9,10,11,12,16]. The additional minute of compression at the same site may allow further polymerisation of the proximal glue plug at the intended level [3,4,5,6,7,10,11,12]. The diameter-based criterion delivers more compression time to larger veins with a greater intraluminal volume [11,12,13]. These remain mechanistic hypotheses, and, as noted above, the three components were applied together and cannot be evaluated separately.

If confirmed, the modification would be simple to implement: it adds 1–2 min at the initial treatment phase, leaves the segmental compression steps unchanged, and requires no additional equipment, staff or capital cost. Optimising superficial venous ablation is clinically relevant because early endovenous ablation of superficial reflux, combined with compression, accelerates venous leg-ulcer healing and increases ulcer-free time compared with compression alone, as shown in the randomised EVRA trial [23]. Although the present study used a single cyanoacrylate system, other n-butyl-2-cyanoacrylate platforms—such as the VenaBlock non-tumescent system with an application guiding light—achieve high occlusion rates with low reported complication rates [24]; whether compression-related migration and the present modification behave similarly across cyanoacrylate formulations and delivery catheters is unknown. We emphasise that the present data do not support a change in clinical practice: they are exploratory technical observations, and any adoption of this modification should await adequately powered randomised evaluation with clinically adjudicated endpoints.

### Limitations

This study has substantial limitations, and its conclusions are correspondingly restricted. First, the total sample of 30 limbs (15 per group) was determined by feasibility rather than by an a priori power calculation and provides very limited precision, particularly for uncommon outcomes; the comparison of deep venous extension (3/15 vs. 0/15) is clearly underpowered, and its confidence interval spans no difference. Second, allocation was sequential and non-randomised, with the two cohorts treated in consecutive calendar periods by the same operators; temporal bias, selection bias, procedural drift and unmeasured confounding therefore cannot be excluded, and operator experience alone does not eliminate temporal confounding. Third, although the cohorts were comparable for every baseline variable recorded, with all standardised differences ≤ 0.31, a study of this size has very little power to detect imbalance; baseline comparability on the variables we happened to record is therefore weak reassurance rather than evidence that the cohorts were exchangeable. Adjustment of the primary outcome for GSV diameter, age, body mass index and smoking did not materially change the estimate (−1.19 cm, 95% CI −1.44 to −0.94), but adjustment in a sample of this size cannot be relied upon to remove confounding. Fourth, the distance at which compression was actually applied was not recorded prospectively. The post hoc inferred compression level was more distal in the modified cohort (5.21 vs. 4.47 cm), but because it is computed from two post-procedural outcome measurements it cannot serve as a baseline covariate or as an adjustment variable; consequently, differences in residual stump length, in proximity to the SFJ and in the distribution across descriptive distance categories, cannot be attributed to the intervention alone, and this residual confounding is not resolved by the analyses presented. Fifth, outcome assessment was neither blinded nor duplicated: measurements were performed live by assessors aware of allocation; ultrasound clips and still images were not retained, so offline re-measurement was impossible and interobserver and intraobserver reliability could not be quantified; measurement bias in a centimetre-scale ultrasound endpoint cannot be excluded; and no claim is made about the precision of these measurements. Sixth, the binary and continuous migration endpoints were recorded separately rather than derived from one another, and they do not classify every limb identically; both are reported, but the binary endpoint rests on a qualitative operator judgement. Seventh, follow-up was limited to one week, which cannot address late endovenous glue-induced thrombosis, stump evolution, recanalisation, delayed migration, recurrence, symptom relief or durability [13,19]. Eighth, this study collected no systematic clinical outcomes: symptom improvement, VCSS, quality-of-life measures, hypersensitivity and foreign-body reactions, phlebitis, deep vein thrombosis, pulmonary embolism and reintervention were not prospectively ascertained, so the absence of recorded events cannot be read as an absence of events. Ninth, management of the three deep venous extension events was at the discretion of the treating surgeon and was not protocolised; two grade III events were treated with an antiplatelet agent rather than therapeutic anticoagulation, which does not correspond to the treatment algorithm proposed with the classification we applied [7] or to reported practice in larger EGIT series [22]. Although body mass index and antithrombotic exposure were recorded in this cohort and no patient was taking an antiplatelet or anticoagulant agent at baseline, both remain important covariates for future work, since obesity influences venous thrombotic risk and the pharmacology of anticoagulation—a meta-analysis of direct oral anticoagulants versus warfarin in obese patients with atrial fibrillation or venous thromboembolism found comparable efficacy and safety for direct oral anticoagulants in this population [25]. Tenth, 70% of treated limbs were CEAP C1 and no patient underwent a documented trial of conservative management before ablation, both of which depart from usual practice and limit generalisability. Finally, the descriptive distance categories and the 5.5 mm diameter criterion are investigator-defined and unvalidated, and the intervention is a three-component bundle whose elements cannot be evaluated separately. Taken together, these limitations mean that the present work supports only the generation of hypotheses for a properly designed randomised trial.

## 5. Conclusions

In this small, single-centre, non-randomised exploratory pilot cohort, a modified ultrasound-guided compression protocol incorporating a brief “release–check–recompress” cycle was associated with a shorter immediate glue migration distance measured from the compression level. The findings are limited to immediate technical and anatomical ultrasound observations with a single one-week assessment; they rest on a surrogate endpoint measured without blinding, are subject to residual confounding from sequential allocation and from an unrecorded compression distance, and do not establish clinical safety, effectiveness, durability, or superiority. The three deep venous extension events observed under the standard protocol were asymptomatic, were graded Cho III and had resolved at one week; no recommendation for a change in practice, and no diameter-based criterion, can be derived from these data. The modification is simple and adds only 1–2 min to the procedure, which makes it worth evaluating formally: adequately powered, multicentre randomised trials with blinded and duplicated outcome assessment, systematic adverse-event surveillance, prospectively recorded compression distances, protocolised management of any EGIT, and follow-up beyond one week are required before any clinical conclusion can be drawn.

## Figures and Tables

**Table 1:** Baseline demographic and clinical characteristics of the study population.

Variable	Standard Protocol (*n* = 15)	Modified Protocol (*n* = 15)	*p* Value
Age, y	46.0 ± 7.9	47.6 ± 7.7	0.58
Male sex, *n* (%)	5 (33.3)	4 (26.7)	>0.99
Body mass index, kg/m^2^	25.5 ± 3.0	26.3 ± 2.0	0.40
GSV diameter, mm	5.10 ± 0.80	5.17 ± 0.82	0.81
Vein diameter > 5.5 mm, *n* (%)	5 (33.3)	3 (20.0)	0.68
Vein depth, cm	2.19 ± 0.30	2.19 ± 0.33	0.95
CEAP class C2, *n* (%)	5 (33.3)	4 (26.7)	>0.99
Limb (right/left), *n*	8/7	7/8	>0.99
Smoking, *n* (%)	5 (33.3)	4 (26.7)	>0.99
Diabetes mellitus, *n* (%)	3 (20.0)	4 (26.7)	>0.99
Thrombophilia, *n* (%)	0 (0.0)	0 (0.0)	>0.99
Prior superficial thrombophlebitis, *n* (%)	0 (0.0)	0 (0.0)	>0.99
Antiplatelet or anticoagulant use at baseline, *n* (%)	0 (0.0)	0 (0.0)	>0.99

Data are mean ± SD or *n* (%). GSV, great saphenous vein; SFJ, saphenofemoral junction. Standardised differences are reported in the text as the primary metric of baseline balance, as *p* values are of limited value in small samples; all were ≤0.31. Treated vein length, vein tortuosity, reflux time and peak reflux velocity, adjunctive procedures and post-procedure mobilisation were not recorded and could not be compared. The post hoc inferred compression level (the sum of residual stump length and migration distance) is reported in the text rather than in this table because it is derived from post-procedural outcome measurements and is not a baseline covariate. *p* values are from an independent *t*-test (continuous) or Fisher’s exact test (categorical).

**Table 2:** Primary and secondary outcomes between the two protocols.

Outcome	Standard Protocol (*n* = 15)	Modified Protocol (*n* = 15)	Change	*p* Value
**Glue migration distance, cm**	1.97 ± 0.51	0.80 ± 0.34	MD −1.17 (95% CI −1.50, −0.85)	<0.0001
**Residual stump length, cm**	2.49 ± 1.27	4.41 ± 0.34	MD +1.92 (95% CI +1.22, +2.62)	<0.001
**Immediate migration, *n* (%)**	7 (46.7; 95% CI 24.8–69.9)	0 (0.0; 95% CI 0.0–20.4)	RD −46.7% (95% CI −71.9, −21.4)	0.006
**One-week migration, *n* (%)**	8 (53.3; 95% CI 30.1–75.2)	2 (13.3; 95% CI 3.7–37.9)	RD −40.0% (95% CI −70.6, −9.4)	0.050
**Deep venous extension, *n* (%)**	3 (20.0; 95% CI 7.0–45.2)	0 (0.0; 95% CI 0.0–20.4)	RD −20.0% (95% CI −40.2, +0.2)	0.22

Data are mean ± SD or *n* (%) with Wilson score 95% CIs for individual proportions. Residual stump length is the distance of the proximal glue edge from the SFJ and is influenced by the level at which compression was applied (Section 3.1). The Change column gives the between-group effect (modified minus standard) as a mean difference (MD, cm) or risk difference (RD, %) with a 95% confidence interval. The confidence interval for deep-vein extension includes zero, indicating no statistically significant difference. Binary migration outcomes are dichotomisations of the continuous migration measurement at the threshold defined in Section 2.3. *p* values are from an independent *t*-test (continuous) or Fisher’s exact test (categorical). CI, confidence interval; SFJ, saphenofemoral junction.

**Table 3:** Individual characteristics of the three limbs with duplex-detected extension into the deep venous system (standard protocol group).

Variable	Case 1	Case 2	Case 3
Age, y	56	46	59
Sex	Female	Female	Male
CEAP class	C2	C2	C2
Body mass index, kg/m^2^	26	24	29
GSV diameter at treatment site, mm	6.0	6.2	6.3
Glue migration from the compression level, cm	2.8	2.4	2.9
Distance of proximal glue edge from the SFJ, cm	0.6	0.7	0.3
Proportion of CFV luminal area involved, %	50	70	60
EGIT grade (Cho et al. [7])	III	III	III
Symptoms attributed to the event	None (asymptomatic; detected on protocol duplex)	None (asymptomatic; detected on protocol duplex)	None (asymptomatic; detected on protocol duplex)
Antithrombotic therapy administered	Rivaroxaban 20 mg once daily	Rivaroxaban 20 mg once daily	Rivaroxaban 20 mg once daily
Duration of antithrombotic therapy	1 month	1 month	1 month
Duplex status of the deep venous extension at one week	Resolved	Resolved	Resolved
Clinical sequelae at last contact	None	None	None

CEAP, Clinical–Etiologic–Anatomic–Pathophysiologic classification; CFV, common femoral vein; EGIT, endovenous glue-induced thrombosis; GSV, great saphenous vein; SFJ, saphenofemoral junction. All three events occurred in the standard protocol group; no deep venous extension was observed in the modified protocol group. EGIT was graded retrospectively from the procedural duplex records according to Cho et al. [7], which classify events by the proportion of the deep venous luminal area occupied (grade I < 25%, grade II 25–49%, grade III 50–74%, grade IV 75–100%); because that classification is area-based, the linear extent of protrusion into the CFV was not recorded. Antithrombotic management was at the discretion of the treating surgeon and was not protocolised: one patient received a direct oral anticoagulant and two received clopidogrel, which is an antiplatelet rather than an anticoagulant agent. This heterogeneity, and the fact that two grade III events were managed without therapeutic anticoagulation, are acknowledged in the Limitations section.

**Table 4:** Distribution of limbs across descriptive distance categories based on the final distance of the proximal glue edge from the SFJ.

Distance of Proximal Glue Edge from SFJ	Standard Protocol, *n* (%)	Modified Protocol, *n* (%)
<2 cm	4 (26.7)	0 (0.0)
2–3 cm	4 (26.7)	0 (0.0)
>3 cm	7 (46.7)	15 (100.0)

SFJ, saphenofemoral junction. The distance categories are descriptive strata only. They were not prospectively validated, have not been shown to correspond to thrombosis risk, and carry no safety or risk interpretation. This distribution is derived from the residual stump length measurements and is influenced by the level at which compression was applied (Section 3.1).

**Table 5:** Local thrombotic complications of endovenous ablation.

Complication	Definition	Reported Frequency	Typical Management
Endothermal heat-induced thrombosis (EHIT)	Thrombus extension from a thermally ablated superficial vein into the adjacent deep vein	~1–3% after RFA/EVLA	Surveillance for low classes; anticoagulation for propagating or higher-class events
Endovenous glue-induced thrombosis (EGIT)	Adhesive/thrombus extension from a cyanoacrylate-treated vein into the deep venous system	~3–6% (higher with large veins or glue near the SFJ)	Surveillance; anticoagulation for symptomatic or propagating thrombus
Superficial thrombophlebitis/phlebitis	Inflammatory thrombosis of the treated superficial vein	~2–20% (often self-limiting)	NSAIDs and compression; occasionally anticoagulation
Deep vein thrombosis (DVT)	Thrombosis within the deep venous system	<1–2%	Therapeutic anticoagulation
Pulmonary embolism (PE)	Embolisation to the pulmonary circulation	Rare (<0.5%)	Therapeutic anticoagulation; rarely fatal

EHIT, endothermal heat-induced thrombosis; EGIT, endovenous glue-induced thrombosis; RFA, radiofrequency ablation; EVLA, endovenous laser ablation; SFJ, saphenofemoral junction; NSAIDs, non-steroidal anti-inflammatory drugs. Frequencies are approximate ranges compiled from published series [7,10,12,15,22]; classification and management thresholds vary between the EHIT and EGIT schemes.

## Data Availability

The data presented in this study are available from the corresponding author upon reasonable request.

## Supplementary Materials

The following supporting information can be downloaded at: https://www.mdpi.com/article/10.3390/jcm15166141/s1, Supplementary Material S1: STROBE Statement—Checklist of items that should be included in reports of cohort studies.

## Abbreviations

The following abbreviations are used in this manuscript:

ANCOVA	Analysis of covariance
CEAP	Clinical, Etiologic, Anatomic, and Pathophysiologic (classification)
CI	Confidence interval
DVE	Deep vein extension
EGIT	Endovenous glue-induced thrombosis
GSV	Great saphenous vein
SD	Standard deviation
SFJ	Saphenofemoral junction
STROBE	Strengthening the Reporting of Observational Studies in Epidemiology
