# Supplementary material for: Impact of a Modified Ultrasound-Guided Compression Protocol on Glue Migration in Endovenous Ablation Therapy: A Single-Centre, Prospective, Exploratory Pilot Comparative Cohort Study
Source: J Clin Med. 2026 Aug 7;15(16):6141. doi: 10.3390/jcm15166141 (PMC13513685; doi:10.3390/jcm15166141)
Supplement: Supplementary file 1 [file jcm-15-06141-s001.zip › jcm-4456606-supplementary.pdf]

Supplementary Material S1

STROBE Statement — Checklist of items that should be included in reports of cohort studies

Manuscript ID jcm-4456606 — "Impact of a Modified Ultrasound-Guided Compression Protocol on Glue Migration in Endovenous Ablation Therapy: A Single-Centre, Prospective, Exploratory Pilot Comparative Cohort Study"

Page numbers refer to the revised manuscript. Section numbers are given in addition, as pagination may change at typesetting.

Item	Recommendation (STROBE)	How addressed in the manuscript	Location
**Title and abstract**			
1	(a) Indicate the study's design with a commonly used term in the title or the abstract	The title identifies the design as a "Single-Centre, Prospective, Exploratory Pilot Comparative Cohort Study". The Abstract describes two sequential, non-randomly allocated cohorts.	p. 1; Title, Abstract
	(b) Provide in the abstract an informative and balanced summary of what was done and what was found	Structured abstract (266 words) under Background/Objectives, Methods, Results and Conclusions. It reports the primary and secondary results with effect estimates and confidence intervals, and states that clinical and thromboembolic outcomes were not systematically ascertained, that follow-up was one week, and that the findings are hypothesis-generating.	p. 1; Abstract
**Introduction**			
2	Background/rationale: explain the scientific background and rationale for the investigation being reported	Cyanoacrylate ablation, proximal glue migration toward the saphenofemoral junction, and endovenous glue-induced thrombosis are introduced, with the compression manoeuvre identified as an under-studied technical step.	pp. 1–2; §1
3	Objectives: state specific objectives, including any prespecified hypotheses	Stated at the end of the Introduction and in Section 2.1: to compare a standard compression protocol with a modified ultrasound-guided protocol using immediate technical and anatomical ultrasound endpoints. The study is explicitly framed as hypothesis-generating rather than confirmatory.	p. 2; §1, §2.1
**Methods**			
4	Study design: present key elements of study design early in the paper	Single-centre, prospective, exploratory pilot comparative cohort study with two sequential non-randomised cohorts. Section 2.1 states that no a priori sample size calculation was performed, that the sample was determined by feasibility, and that the study was not designed or powered to evaluate clinical effectiveness, durability or safety.	p. 2; §2.1
5	Setting: describe the setting, locations, and relevant dates, including periods of recruitment, exposure, follow-up, and data collection	Single centre. Enrolment dates are given separately for each cohort (standard, 10 January to 25 February 2026; modified, 28 February to 20 April 2026). Assessments were performed immediately after the procedure and at one week. Operator distribution across cohorts is reported.	pp. 2–3; §2.1
6	(a) Participants: give the eligibility criteria, and the sources and methods of selection of participants; describe methods of follow-up	Eligibility and exclusion criteria are listed in full, including CEAP class, GSV diameter range and reflux duration. Thirty consecutive eligible patients were enrolled from 50 assessed. The symptoms recorded in CEAP C1 patients and the exclusion of alternative causes are described; it is stated explicitly that no trial of conservative management preceded ablation. Follow-up was clinical and duplex assessment immediately post-procedure and at one week.	pp. 3–5; §2.1, §2.2
	(b) For matched studies, give matching criteria and number of exposed and unexposed	Not applicable — this was not a matched study. Allocation was by sequential cohort (15 limbs per group).	—
7	Variables: clearly define all outcomes, exposures, predictors, potential confounders, and effect modifiers; give diagnostic criteria, if applicable	The primary outcome (glue migration distance from the compression level), the principal anatomical safety measure (residual stump length, referenced to the SFJ), and the three binary secondary outcomes are each defined. Deep venous extension was graded by the classification of Cho et al. Section 2.2 distinguishes catheter-tip position, glue-delivery point, transducer position and compression level to avoid ambiguity.	pp. 5–8; §2.2, §2.3
8	Data sources/measurement: for each variable of interest, give sources of data and details of methods of assessment; describe comparability of assessment methods if there is more than one group	Duplex ultrasound within five minutes of catheter removal and again at one week; longitudinal plane, electronic callipers, measured from the proximal margin of the glue cast to a skin marking placed over the compression level; one measurement per limb. The same two	pp. 7–8; §2.3

		experienced sonographers and the same standardised protocol were applied to both cohorts.	
**9**	Bias: describe any efforts to address potential sources of bias	Sources of bias are described rather than claimed to be eliminated. Section 2.1 states that sequential non-randomised allocation leaves temporal confounding, selection bias and procedural drift unaddressed. Section 2.3 states that assessors were unblinded and that neither interobserver nor intraobserver reliability was quantified, because imaging was not retained for offline re-measurement. Section 2.4 explains why the derived compression level cannot be used as an adjustment variable without circularity. Covariate adjustment (GSV diameter, age, body mass index, smoking) is reported as partial mitigation only.	pp. 2–3, 7–9; §2.1, §2.3, §2.4
**10**	Study size: explain how the study size was arrived at	Stated in Section 2.1: no formal a priori sample size calculation was performed; 15 limbs per group was determined by feasibility. Sample size projections for a future confirmatory trial are given at the end of the same section.	p. 2; §2.1
**11**	Quantitative variables: explain how quantitative variables were handled in the analyses; if applicable, describe which groupings were chosen and why	Continuous variables were analysed as continuous. Descriptive distance categories (< 2 cm, 2–3 cm, > 3 cm) are stated to be arbitrary, investigator-defined strata chosen for readability, not validated thresholds, and carrying no risk or safety interpretation. The 5.5 mm diameter criterion is described as a component of the intervention, not as an analytic grouping.	pp. 8–9, 16; §2.3, §2.4, §3.4
**12**	(a) Statistical methods: describe all statistical methods, including those used to control for confounding	Shapiro–Wilk for normality (with results reported, including non-normality of residual stump length in the standard cohort); independent-samples t-tests; Mann–Whitney U as sensitivity analysis; Fisher's exact test; ANCOVA adjusting first for GSV diameter and then additionally for age, body mass index and smoking. Software and versions are given.	pp. 8–9; §2.4
	(b) Describe any methods used to examine subgroups and interactions	Stated explicitly that no diameter-stratified subgroup analysis is presented and no treatment-by-diameter interaction was tested, because the stratum of veins > 5.5 mm contained only eight limbs in total.	p. 9; §2.4, §3.2
	(c) Explain how missing data were addressed	There were no missing data for any recorded variable; all 30 enrolled limbs contributed complete data for every analysed outcome. Variables that were never collected are listed explicitly (treated vein length, vein tortuosity, reflux time and peak reflux velocity, adjunctive procedures, post-procedure mobilisation) and are not imputed.	pp. 9–10; §3.1
	(d) If applicable, explain how loss to follow-up was addressed	No participant was lost to follow-up: all 30 patients attended the one-week assessment. Patients unable to attend follow-up duplex were excluded before enrolment (Figure 1).	pp. 3–4; §2.1, Figure 1
	(e) Describe any sensitivity analyses	A prespecified sensitivity analysis re-derives the immediate migration endpoint from the continuous measurement at a ≥ 2.0 cm threshold and is reported alongside the prospectively recorded endpoint. The Mann–Whitney U test is reported as a non-parametric sensitivity analysis for continuous outcomes.	pp. 8–9, 12–15; §2.3, §2.4, §3.3
**Results**			
**13**	(a) Participants: report numbers of individuals at each stage of the study	50 patients assessed for eligibility; 20 excluded; 30 enrolled and allocated 15 per cohort; 30 completed immediate and one-week assessment.	pp. 3–4; §2.1, Figure 1
	(b) Give reasons for non-participation at each stage	Reasons for the 20 exclusions are listed: GSV diameter outside the specified range, prior venous intervention, acute thrombosis, inability to attend follow-up duplex, and refusal of consent.	p. 3; §2.1, Figure 1
	(c) Consider use of a flow diagram	Provided as Figure 1.	p. 4; Figure 1
**14**	(a) Descriptive data: give characteristics of study participants and information on exposures and potential confounders	Table 1 reports age, sex, body mass index, GSV diameter, proportion of veins > 5.5 mm, vein depth, CEAP class, treated limb, smoking, diabetes, thrombophilia, prior superficial thrombophlebitis and baseline antiplatelet/anticoagulant use, with standardised differences reported in the text.	pp. 9–11; §3.1, Table 1
	(b) Indicate number of participants with missing data for each variable of interest	No missing data for any recorded variable (n = 15 per group for every entry in Table 1). Variables not collected are identified as such rather than reported as missing.	pp. 9–11; §3.1, Table 1

	(c) Summarise follow-up time (eg, average and total amount)	Follow-up was a fixed single interval of one week for all 30 participants; no longer follow-up was undertaken. This is stated in the Methods, Results, Limitations and Conclusions.	pp. 7, 12–15, 20; §2.3, §3.3, §4.1
**15**	Outcome data: report numbers of outcome events or summary measures over time	Continuous outcomes with means, standard deviations and ranges; binary outcomes as counts and percentages with Wilson score 95% confidence intervals. Immediate technical occlusion 15/15 in each group. Table 3 gives patient-level data for all three deep venous extension events.	pp. 11–15; §3.2, §3.3, Tables 2 and 3
**16**	(a) Main results: give unadjusted estimates and, if applicable, confounder-adjusted estimates and their precision; make clear which confounders were adjusted for and why they were included	Unadjusted mean difference −1.17 cm (95% CI −1.50 to −0.85); adjusted for GSV diameter −1.20 cm (95% CI −1.47 to −0.92); further adjusted for age, body mass index and smoking −1.19 cm (95% CI −1.44 to −0.94). The rationale for each covariate, and the reason the derived compression level was excluded from adjustment, are stated.	pp. 8–9, 11–12; §2.4, §3.2
	(b) Report category boundaries when continuous variables were categorized	Boundaries are given for the descriptive distance categories (< 2 cm, 2–3 cm, > 3 cm), for the diameter strata (≤ 5.5 mm, > 5.5 mm) and for the sensitivity-analysis migration threshold (≥ 2.0 cm), each with a statement that they are investigator-defined and unvalidated.	pp. 8–9, 12, 16; §2.3, §3.2, §3.4
	(c) If relevant, consider translating estimates of relative risk into absolute risk for a meaningful time period	All binary comparisons are reported as absolute risk differences with 95% confidence intervals rather than relative measures, over the fixed one-week observation period. Relative-change percentages were removed from Table 2 during revision as potentially misleading.	pp. 12–14; §3.3, Table 2
**17**	Other analyses: report other analyses done — eg analyses of subgroups and interactions, and sensitivity analyses	The sensitivity analysis of the immediate migration endpoint is reported in full. A post-hoc descriptive observation on the derived compression level, including its distribution and the four standard-cohort limbs with implausibly proximal values, is reported with an explicit statement that it is not a baseline covariate. No subgroup or interaction analyses were performed.	pp. 9–10, 12–15; §3.1, §3.3
**Discussion**			
**18**	Key results: summarise key results with reference to study objectives	The first Discussion paragraph summarises the primary and secondary findings against the stated objective, including the characterisation of the three deep venous extension events.	pp. 17–18; §4
**19**	Limitations: discuss limitations of the study, taking into account sources of potential bias or imprecision; discuss both direction and magnitude of any potential bias	A dedicated Limitations section sets out eleven numbered limitations, including the absence of a power calculation, sequential non-randomised allocation, the limited power of the baseline comparison, the unrecorded compression distance and its consequences for SFJ-referenced outcomes, unblinded and unduplicated outcome assessment, the qualitative basis of the binary endpoint, one-week follow-up, the absence of systematic clinical outcome ascertainment, non-protocolised management of the EGIT events, the high proportion of CEAP C1 limbs with no preceding conservative management, and the unvalidated investigator-defined categories. The direction of bias is stated where it can be inferred.	pp. 20–22; §4.1
**20**	Interpretation: give a cautious overall interpretation of results considering objectives, limitations, multiplicity of analyses, results from similar studies, and other relevant evidence	The Discussion and Conclusions state that the findings rest on an unblinded surrogate endpoint, are subject to residual confounding, and establish neither clinical safety nor effectiveness, durability or superiority. P values are interpreted descriptively without multiplicity adjustment, as stated in Section 2.4. Results are placed against the conflicting published evidence on vein diameter and EGIT risk, including the divergence of our own observations from those of Cho et al.	pp. 9, 17–19, 22; §2.4, §4, §5
**21**	Generalisability: discuss the generalisability (external validity) of the study results	Discussed explicitly: a single-centre Middle Eastern cohort in which 70% of treated limbs were CEAP C1 and no patient underwent a trial of conservative management, which is stated to sit outside both the population and the care pathway in which truncal ablation is most commonly performed. Regional variation in varicose-vein epidemiology is discussed with reference to the literature.	pp. 18–19, 21; §4, §4.1
**Other information**			
**22**	Funding: give the source of funding and the role of the funders for the present study and, if applicable, for the original study on which the present article is based	Stated in the Funding declaration at the end of the manuscript.	p. 23; Funding statement
